# Maternal-Offspring Interactions: Reciprocally Coevolving Social Environments

**DOI:** 10.1093/jhered/esab044

**Published:** 2021-11-30

**Authors:** Michael J Wade

**Affiliations:** Department of Biology, Indiana University, Bloomington, IN 47405, USA

**Keywords:** epistasis, genotype-by-environment interaction, indirect genetic effects, inbreeding, Price Equation

## Abstract

Maternal-zygotic co-evolution is one of the most common examples of indirect genetic effects. I investigate how maternal-zygotic gene interactions affect rates of evolution and adaptation. Using comparably parameterized population genetic models, I compare evolution to an abiotic environment with genotype-by-environment interaction (G × E) to evolution to a maternal environment with offspring genotype-by-maternal environment interaction (G × G_maternal_). There are strong parallels between the 2 models in the components of fitness variance but they differ in their rates of evolution measured in terms of ∆p, gene frequency change, or of ∆W, change in mean fitness. The Price Equation is used to partition ∆W into 2 components, one owing to the genetic variance in fitness by natural selection and a second owing to change in environment. Adaptive evolution is faster in the 2-locus model with G × G_maternal_ with free recombination, than it is in the 1-locus model with G × E, because in the former the maternal genetic environment coevolves with the zygotic phenotype adapting to it. I discuss the relevance of these findings for the evolution of genes with indirect genetic effects.

Maternal-zygotic co-evolution is one of the best examples of why so many features of adaptations involve and are dependent upon indirect genetic effects. I would argue that there are no aspects of adaptive evolution of complex phenotypes that do not involve maternal indirect genetic effects owing to their critical involvement in the earliest stages of development. Mothers are an ubiquitous, essential component of the capacity of living systems to reproduce. Whether we are considering the reproductive fissioning of a mother cell giving rise to daughter cells in a single celled protist or the more complex, sex-specific multi-cellular reproductive process of a eukaryote, maternal genetic information is an essential feature of the reproductive process.

In all eukaryotes, messenger RNA transcripts from genes in the maternal genome control early offspring development. These maternal messenger RNAs are sequestered nonrandomly in the egg prior to its fertilization and, post-fertilization, they guide early development. Maternal gene products turn on gene expression in the zygote and, at a point in development, called the maternal-zygotic transition, control of embryo development is passed from maternal genes with indirect effects to zygotic genes with direct effects. Many genes have *both* maternal and zygotic transcripts, but some, like genes *biocid* in flies and *mater* in the mouse, are genes with strictly maternal effects acting on the zygotic phenotype. Selection on strictly maternal effect genes depends upon offspring fitness rather than maternal fitness. As a result, death of an offspring caused by a gene in the maternal genome is less effective at causing genetic change than is the death of an offspring caused by a gene with a direct effect in its own genome (Fitzpatrick and Wade 2021). As a result, within populations, strictly maternal effect genes tend to be more polymorphic than other genes in the same gene family that have zygotic expression and have similar effects on offspring fitness (Barker et al. 2005; Cruickshank and Wade 2008; Wade et al. 2009). The weaker selection on strictly maternal effect genes also results in their having a *relatively* greater rate of diversification among species (Demuth and Wade 2007; Cruickshank and Wade 2008). Similar patterns of polymorphism within species and *relative* divergence among them characterize other genes with indirect effects, such as genes with exclusively sib-social effects (Linksvayer and Wade 2009) and genes whose expression is subject to control by quorum sensing (Van Dyken and Wade 2010). In this way, maternal effect genes are also similar to genes whose expression is dependent on environmental condition (Van Dyken and Wade 2010) or sex-limited in their expression (e.g., sperm protein genes, Dapper and Wade 2020).

In addition to genes, mothers transmit organelles and intra- and extra-cytoplasmic microbes to their offspring. One of the best examples of this kind of transmission comes from the studies of dung beetles from the laboratory of my colleague, Armin Mozeck (Parker et al. 2019). The female dung beetle lays her egg within the dung ball on a pedestal made of her waste and microbes. Hatching larvae eat the pedestal and it contributes to their successful early larval development. If deprived of the pedestal or given a pedestal from another species of dung beetle, larval development is impaired. Maternal transmission of obligately tended mealy bugs occurs in the ant genus, Acropyga, where alate queens grasp a mealy bug in their mandibles before undertaking a mating flight (Blaimer et. al. 2016). Moreover, mealy bugs depend upon nutritional symbionts (McCutcheon and Von Dohlen 2011) which are part of this co-dispersing multi-species community.

My central question is this: how do maternal-zygotic interactions affect evolution and adaptation? And in particular, I am interested in the question: *What is the evolutionary difference between adaptation to an abiotic environment with genotype-by-environment interaction (G x E) and adaptation to a maternal environment with offspring genotype-by-maternal environment interaction (G x G*_*maternal*_*)?* A similar question could be asked in terms of adaptation of a host to its symbionts when there is symbiont-by-host genotype environment interaction (G_Host_ × G_Symbiont_). In this paper, I will use the classical 1 locus-2 allele model of G × E and contrast its features with those of the 2-locus population genetic model of G × G_maternal_ from Drown and Wade (2014). The 2 types of interaction will be parameterized in the same way with respect to fitness.

In outline, I first introduce the basic model and its parameterization. Second, I partition the total variance in fitness into its 3 components for both models: the genetic variance, the environmental variance and the interaction variance. This will facilitate model comparison.

Third, I will point out the relationship between the variance components and gene frequency change, ∆p, and contrast the rates of gene frequency evolution in the 2 models. Fourth, I will examine the relationship between the 3 variance components and the change in mean fitness, ∆W. To do this, I will use the Price equation (Frank 2012) to illustrate the difference in how mean fitness changes with adaptation to an abiotic environment with G × E versus adaptation to a biotic environment with G × G_maternal_. Whether measured by ∆p or ∆W, adaptation to a biotic environment with G × G_maternal_ can be much more rapid than adaptation to an abiotic environment with G × E (Wolf et al. 1998; Drown and Wade 2014).

## The Models: G × E, G × G_maternal_ and Norms of Reaction

Genotypic reaction norms and G × E are central concepts in the evolution of phenotypic plasticity as they quantify the amount of variation from one genotype to another in the response to environmental variation. The reaction norm, a plot of mean phenotype versus an environmental variable, is way of illustrating G × E (Falconer 1952, 1990; Schlichting and Pigliucci 1998). Visually, G × E is seen in a reaction norm plot as a set of non-parallel lines, one for each genotype when that genotype is reared across a range of environments. When the lines are parallel, although one genotype may differ from another, all genotypes respond in the same way to change in the environment.

Reaction norms and genetic correlations are different ways of depicting G × E. Statistically, G × E can be viewed as a genetic correlation between two traits, a trait measured in one environment and the same trait measured in a second environment (Falconer 1952, 1990).

Genotypes that vary little in fitness or other phenotypic effect with change in an environment are often contrasted with “plastic genotypes” that vary a great deal in phenotype with change in environment (Taylor et al. 2019). An extreme type of plasticity, polyphenism, is the production of alternative phenotypes induced by different environmental conditions. A central and still controversial question in evolutionary biology concerns the role of G × E in the evolution of adaptive plasticity. Direct selection for plasticity implies that it is adaptive and that there may be genes for plasticity (plasticity genes; Callahan et al. 2005). An alternative view is that all selection for plasticity is indirect and plasticity arises as an evolutionary byproduct of adaptive differential gene regulation in different environments. Maternal genetic effects may involve both direct and indirect selection at the among-family level for plasticity. And, many polyphenisms in nature have been shown to be under maternal genetic control as opposed to zygotic genetic control (Dury and Wade 2020).

**Figure 1.**
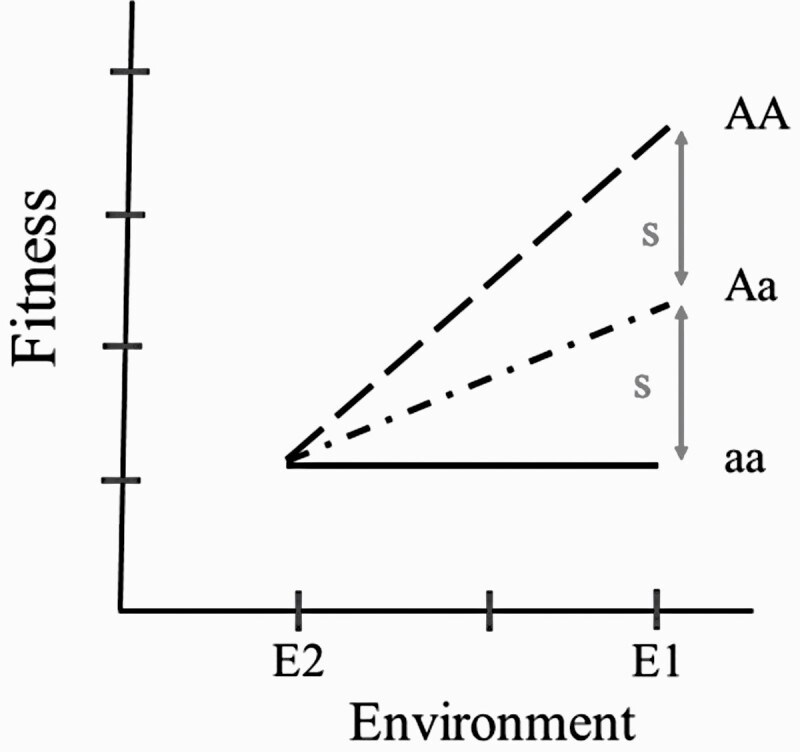
The norms of reaction for each of the 3 A-locus genotypes in the G x E model. These genotypes have the same fitness in E2 but differ in fitness in E1. The fitness of genotype aa is invariant, but that of AA is adaptive in E1.

The G × E model (Figure 1) that I introduce here is based on a simple one-locus 2-allele randomly mating population genetic model, with alleles A and a, in frequencies p_A_ and q_a_, respectively. For ease of comparison, I use scale G × E, one of the 2 categories of G × E. Scale G × E occurs when the differences between genotypes change in magnitude with change in the environment but do not change in rank. The evolutionary implication is that the same gene favored in one environment is also favored in another, although the magnitude of the effect differs between environments. Animal breeders *define scale G x E* (James 2009, p. 152 my emphasis) in this way, “***In a stricter sense, a scale-type interaction is one which can be removed by transformation of the scale of measurement****, but many cases in which such a scale transformation has not been identified can usefully be regarded as being of scale type. On the other hand, rank-type interactions are those in which genotype A may be superior to genotype B in environment 1, but the reverse is true in environment 2. The importance of this distinction is obvious, since with scale-type interactions a genotype selected as best in one environment will be best in the other environments considered, only the magnitude of the superiority being affected, while with rank-type interactions the best genotype selected in one environment may perform poorly in another. Thus, the distinction between these types of G × E has important implications for the design of breeding programs*.” Here, I use scale G × E instead of rank G × E in order to introduce a new approach to illustrating and quantifying a fundamental aspect of indirect genetic effects, namely, that such effects permit the environment to coevolve. As I show below, indirect genetic effects manifest themselves in the second term of the Price equation, the environmental term causing change in mean fitness. The derivation below shows that the less interesting scale G × E, becomes much more evolutionarily interesting with epistasis between direct and indirect effects, i.e., scale G × G.

No transformation of scale can remove rank G × E, wherein the genotype with the highest fitness in one environment has lowered fitness in another. Scale G × E does not lead to polymorphism at equilibrium, while, with rank G × E, it is possible depending on the amount and frequency of gene flow between environments. Here, I will assume that there is complete gene flow between environments at each generation in order to assume later that, owing to random mating, there is mixing or free recombination of maternal and offspring genotypes, when I replace the 2 abiotic environments with the indirect genetic effects of the 3 maternal genotypes. I use the simpler scale G × E here in order to illustrate the difference between G × E and G × G in both gene frequency change and mean fitness change. This same approach applied to rank G × E greatly changes the degree of difficulty of the algebra, but not the major points that I make below.

The y axis of Figure 1 is the fitness of a genotype and the x-axis represents 2 environments. Environment E1 is a selective environment; that is, in this environment the 3 possible genotypes, AA, Aa, and aa, differ from one another in fitness and in plasticity. Environment E2 is a nonselective environment: all genotypes have the same fitness in environment E2 see Figure 1).

The average fitness for each genotype is its mean fitness across the 2 different environments. Let G_i_ be the frequency of the i-th genotype (i = 1, 2, 3); let W_i,j_ be the fitness of the i-th genotype in the j-th environment (j = 1, 2); and, let f_j_ be the frequency of environment j. If genotype, AA, is genotype 1, its average fitness across both environments is W_1,._ = f_1_W_1,1_ + f_2_W_1,2_. The fitness of every genotype is a function of the frequency of the selective environment. The selection coefficient, s, is positive as depicted. The mean fitness of AA homozygotes is (1 + 2f_1_s), that of Aa heterozygotes is (1 + f_1_s), and that of aa homozygotes (1). The selection coefficient (f_1_s) is a function of the frequency of the selective environment E1. The effect of the A allele on fitness is additive, incrementing genotypic fitness by (f_1_s) for each A allele in an individual’s genotype.

**Figure 2.**
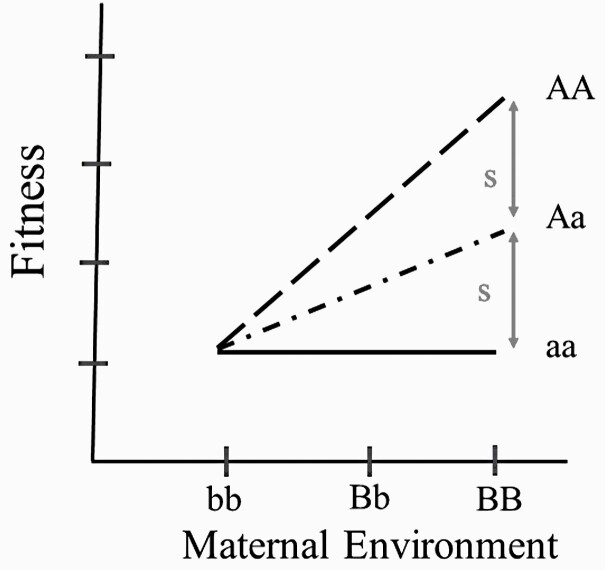
The norms of reaction for each of the 3 A-locus genotypes in the G × G model. These genotypes have the same fitness if the maternal genotype is bb but differ in fitness when the maternal genotype is BB. The fitness of genotype aa is invariant, while that of AA is adaptive when the maternal genotype is Bb or BB.

I parameterize the maternal-zygotic model of G × G_maternal_ in a way comparable to that of the G × E model (Figure 2). There are 2 genes in this epistatic model, the offspring A locus with alternative alleles A and a, in frequencies p_A_ and q_a_, respectively, and the maternal B locus with alternative alleles B and b, in frequencies p_B_ and q_b_, respectively. Offspring of bb mothers have equal fitness regardless of offspring genotype. That is, from the viewpoint of the A locus expressed by offspring, the bb maternal genotypic background is a nonselective background similar to E2 above. However, when the maternal genotype is either Bb or BB, then there are fitness differences among the 3 offspring A-locus genotypes as shown in Figure 2. With both models parameterized in a comparable way (compare Figures 1 and 2), I now turn to the components of the variance in fitness.

## Partitioning Fitness Variance Into Components

In Table 1, I provide another way of reporting the changes in genotypic fitness that attend a changing environment. Here, the column means give you the main effect of each environment and the row means are the genotypic fitness means. Because the main effect of E1 is different from the main effect of E2, there is environmental variance, V_E_. Because the genotypic fitness means differ, there is genetic variance, V_G_. Lastly, because there is an interaction between genotype fitness and environment (Figure 1), there is interaction variance, V_GxE_.

**Table 1.** Genotypic fitness as a function of the environment

	Environment		
Offspring Genotype	E1	E2	Row Mean
AA	1 + 2s	1	1 + 2sf_1_
Aa	1 + s	1	1 + sf_1_
aa	1	1	1
Column Mean	1 + 2sp_A_	1	1 + 2sf_1_p_A_

Using the values in Table 1, we use the grand mean, (1 + 2sf_1_p_A_), and cell entries to calculate the total variance in fitness, V_Total_. The components are calculated using the grand mean and the genotypic means (rows) for V_G_, the grand mean and the environmental means (columns) for V_E_, and the genetic correlation across environments for V_GxE_. When we do this, we find the following:


$$\begin{array}{l} V_{Total}=\;V_{G}+\;V_{E}+\;V_{GxE} \end{array}$$ (1)

where


$$\begin{array}{l} V_{G}=\;{\left(f_{1}s\right)}^{2}\left(2p_{A}q_{a}\right) \end{array}$$ (2a)

$$\begin{array}{l} V_{E}=\;{\left(2sp_{A}\right)}^{2}\left(f_{1}f_{2}\right) \end{array}$$ (2b)

$$\begin{array}{l} V_{GxE}=\;{\left(s\right)}^{2}\left(2p_{A}q_{a}\right)\left(f_{1}f_{2}\right). \end{array}$$ (2c)

In Table 2, the genotypic fitnesses at the A locus that attend a changing maternal genotypic environment are given. As above, the column means give the main effect of each maternal genotypic environment and the row means are the offspring genotypic fitness means. Because the main effects of bb, Bb and BB are different from one another, the maternal genotype functions as the environmental variance, V_E_ = V_GB_. Because the offspring genotypic fitness means differ, there is genetic variance, V_GA_. Lastly, because there is an interaction between offspring genotype fitness and maternal genotypic environment (Figure 2), there is interaction variance, V_GxG_.

**Table 2.** Genotypic fitness as a function of the maternal genotypic environment

	Maternal genotypic environment			
Offspring genotype	BB	Bb	bb	Row Mean
AA	1 + 2s	1 + s	1	1 + 2sp_B_
Aa	1 + s	1 + 0.5s	1	1 + sp_B_
aa	1	1	1	1
Column Mean	1 + 2sp_A_	1 + sp_A_	1	1 + 2sp_A_p_B_

Using the values in Table 2, we use the grand mean, (1 + 2sp_A_p_B_), and cell entries to calculate the total variance in fitness, V_Total_. The components are calculated using grand mean and the genotypic means (rows) for V_GA_, the grand mean and the environmental means (columns) for V_GB_, and the remainder is V_GxG_. When we do this, we find the following:


$$\begin{array}{l} V_{Total}=\;V_{GA}+\;V_{GB}+\;V_{GxG} \end{array}$$ (3)

where


$$\begin{array}{l} V_{GA}=\;{\left(sp_{B}\right)}^{2}\left(2p_{A}q_{a}\right) \end{array}$$ (4a)

$$\begin{array}{l} V_{GB}=\;{\left(sp_{A}\right)}^{2}\left(2p_{B}q_{b}\right) \end{array}$$ (4b)

$$\begin{array}{l} V_{GxE}=\;{\left(s\right)}^{2}\left(2p_{A}q_{a}\right)\left(2p_{B}q_{b}\right). \end{array}$$ (4c)

When we compare Equations [2] and [4], it is clear that the frequency of the selective environment, f_1_, in Equation [2] has been replaced by p_B_, the frequency of the maternal B allele. In addition, the environmental variation term, (f_1_f_2_), in Equation [2] has been replaced by the maternal genic variation term, 2p_B_q_b_, in Equation [4]. I will now turn to the expressions for gene frequency change, ∆p_A_, and mean fitness change, ∆W, for these 2 similarly parameterized models.

## The Rates of Gene Frequency Change With G × E and G × G

### The Rate of Change in p_A_ with G × E

The rate of gene frequency change for the G × E model of Figure 1 and Table 1 is


$$\begin{array}{l} \Delta p_{A}=\;\left(sf_{1}\right)\left(p_{A}q_{a}\right)/W_{GxE}, \end{array}$$ (5)

where mean fitness, W_GxE_ equals (1 + 2sf_1_p_A_) and there is complete gene flow among environments at the start of each generation. The genic effect on fitness is (sf_1_) and the genic variance is (p_A_q_a_) or ½ of the genotypic variance (Walsh and Lynch 2018, chapter 14). Change in A allele frequency depends on the strength of the genic effect on fitness which is clearly a function of f_1_, the frequency of the selective environment. The *environmental effect* is a concept analogous to *genic effect* and, here, it equals (sp_A_). With G × E, the genic and environmental effects are inter-dependent, with one being a frequency of the abundance of the other.

**Figure 3.**
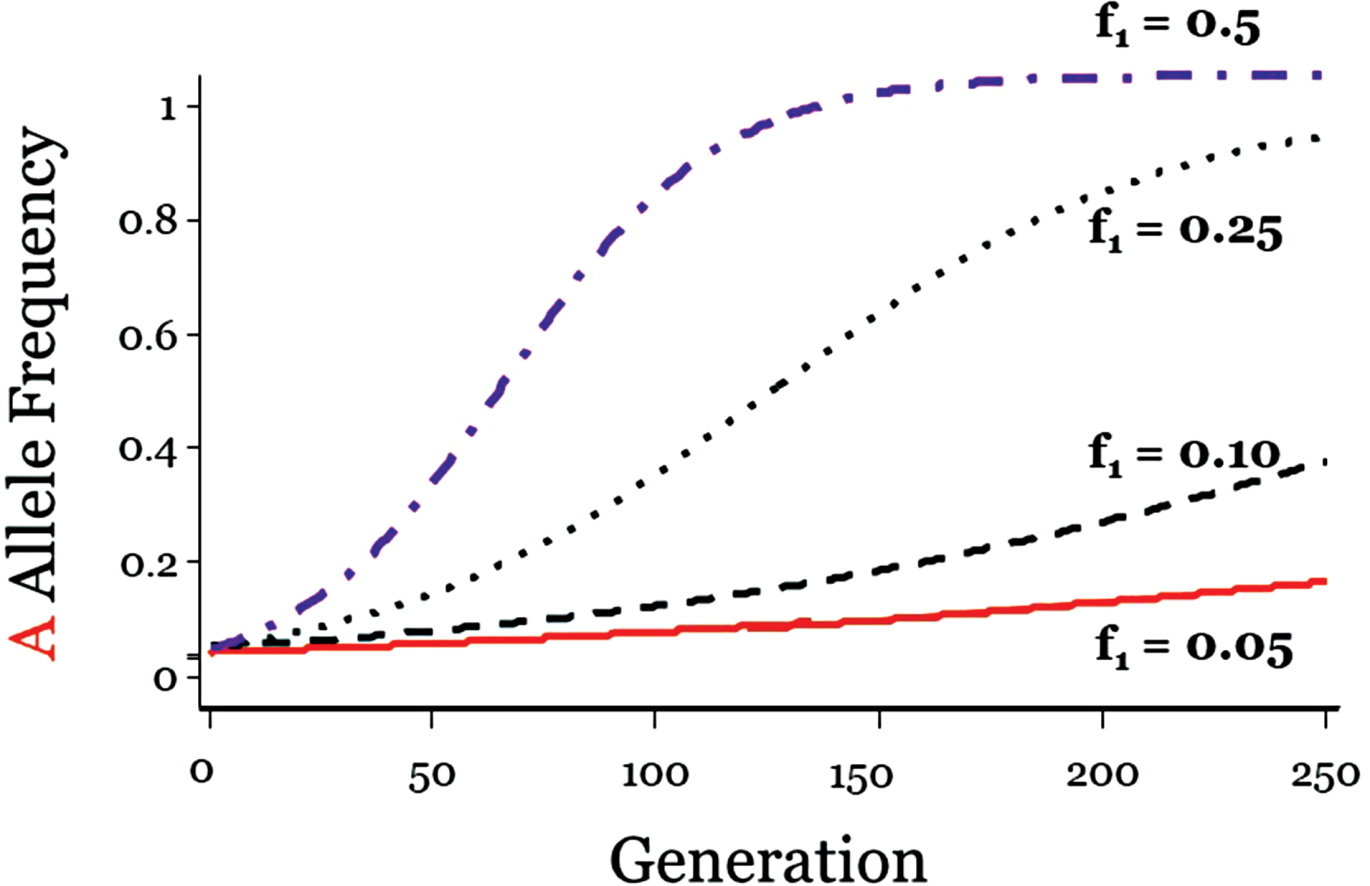
The frequency of p_A_ for 4 different frequencies of E_1_, the selective environment: f_1_ = 0.05, 0.10, 0.25, and 0.050. The rate of evolution of p_A_ accelerates as the frequency of the selective environment becomes larger. (After Drown and Wade 2014).

From Equation [5], it is clear that the rate of adaptation depends on whether E1, the selective environment, is rare or common (Figure 3). Evolution is slow when E1 is rare (f_1_ near 0) because selection is weak. Conversely, evolution is relatively faster when E1 is common (f_1_ near 1). Note that, it is assumed that f_1_, the frequency of the selective environment, remains constant throughout the period of adaptive evolution.

### The Rate of Change in p_A_ With G × G

Here, I assume that the A and B loci are unlinked and, that selection is sufficiently weak that it does not create lasting linkage disequilibrium (LD), so that I can treat LD as 0. This assumption will be relaxed below. The rates of gene frequency change for the G × G model of Figure 2 and Table 2 are


$$\begin{array}{l} \Delta p_{A}=\;\left(sp_{B}\right)\left(p_{A}q_{a}\right)/W_{GxG}, \end{array}$$ (6a)

$$\begin{array}{l} \Delta p_{B}=\;\left(sp_{A}\right)\left(p_{B}q_{b}\right)/2W_{GxG} \end{array}$$ (6b)

where mean fitness, W_GxG_ equals (1 + 2sp_B_p_A_). If there were no maternal-zygotic epistasis, then W would equal (1 + 2sp_A_ + 2s_m_p_B_), where s and s_m_ were the independent effects of the offspring allele A and the maternal allele B on offspring viability. Without epistasis, the term (sp_B_) in Equation [6a] would be simply s, the effect of the A allele independent of maternal genotype. Similarly, without epistasis, the term (sp_A_) in Equation [6b] would be a constant s_m_, the effect of the maternal B allele on offspring viability. The factor ½ is important in Equation [6b] because the change in the frequency of allele B caused by the death of an offspring changes via the maternal-offspring genetic regression, which is ½ with random mating. Change in A allele frequency depends on the strength of the genic effect on fitness which is clearly a function of p_B_, the frequency of the selective maternal genetic environment. The *environmental effect* here is the effect of the *maternal genetic background* which equals (sp_B_). With G × G, the genic and environmental effects are also inter-dependent, with one being a frequency of the abundance of the other. Unlike the G × E case, where ∆f_1_ = 0, here the evolutionary description is incomplete without an additional equation for the genetic covariance between loci A and B, the linkage disequilibrium (LD). The value of LD is increased by selection on AB gene combinations and decreased by recombination. We will investigate these effects on LD in the sections below.

**Figure 4.**
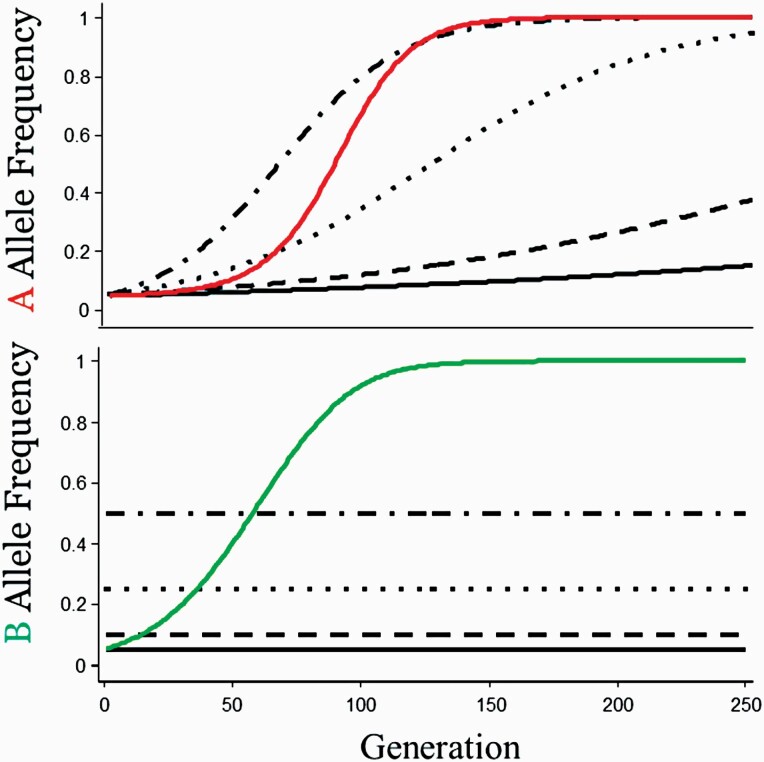
The frequency of p_A_ (upper panel) and p_B_ (lower panel) over time from the initial starting point p_A_ = p_B_ = 0.05. The 4 dashed lines in both graphs are the 4 different frequencies of E_1_, the selective environment: f_1_ = 0.05, 0.10, 0.25, and 0.050 of Figure 3. The rate of evolution of p_A_ accelerates as p_B_ increases. Unlike the unchanging frequencies of E_1_ (lower panel, horizontal lines), the frequency of p_B,_ the maternal genetic environment, co-evolves with the offspring A allele which is adapting to it. (After Drown and Wade 2014).

From Equation [6], it is clear that the rate of adaptation of the zygotic allele, A, depends on whether the B allele is rare or common in mothers (Figure 4). However, this is where the models of G × E and G × G part company. With G × E, the rate of evolution depends entirely on the initial constant value of f_1_ (Figure 3). Here, although the p_B_ allele might be rare initially (p_B_ = 0.05), Equation [6b] guarantees that it will increase as allele A increases. With G × G, the environment itself evolves. Compare the rate of evolution of the A allele (starting at p_A_ = 0.05) when E_1_ has an initial frequency of 0.05 with the rate of evolution of the A allele when the maternal genetic environment has an initial starting point of p_B_ = 0.05. The trajectory of p_A_ with G × G is *faster* than it is with G × E taken from comparable starting points (Figure 4, upper panel, compare solid black line with the red line). For the maternal genetic environment case, p_A_ fixes in approximately the same time as the case where the selective abiotic environment is initially 5 times more common, f_1_ = 0.50 (Figure 4, upper panel, compare dash-dot line with the red line). Adaptation to a biotic environment (i.e., maternal genetic effect) can be much faster than adaptation to a abiotic environment, because the maternal genetic environment does not remain at its initial frequency of 0.05 as does the frequency of E_1_ (Figure 4, lower panel, compare solid black line with the green line). Evolution might appear slower in Equation [6b] since the selection coefficient is not s but rather sp_B_ where p_B_ is initially far from 1. Every generation however, the selection coefficient experienced by the A allele changes from (sp_B_) to (s[p_B_ + ∆p_B_]) and, for the type of epistasis represented here, ∆p_B_ > 0. Thus, the strength of selection on the A allele increases at every generation owing to its co-evolving maternal genetic environment.

### The change in W, mean fitness: a view from the Price Equation

Evolution can also be viewed as ∆W, change in the mean fitness of a population before (W) and after (W’) selection, instead of or in addition to change in gene frequency. The Price Equation (Frank 2012) is a general method for decomposing ∆W into its components. It is particularly revealing of the difference between the process of adaptation to an abiotic environment (G × E model) and that of adaptation to a biotic environment (G × G model). The general formula equals


$$\begin{array}{l} \Delta W\;=\;(W\text{'}|E\text{'})\;-\;\left(W|E\right) \end{array}$$ (7a)

$$\begin{array}{l} \Delta W\;=\;(W\text{'}\left|E\;-\;W\right|E)\;+\;(W\text{'}|E\text{'}\;W\text{'}|E) \end{array}$$ (7b)

where (W’|E) is mean fitness *after selection* with the environment held constant, while (W’|E’) is the mean fitness *after selection* accounting for any change in the environment (E’) that might attend selection.

The partitioning of ∆W in Equation [7b] has a straightforward interpretation. The first term on the righthand side, (W’|E - W|E), is the change in mean fitness owing to natural selection. The second term, (W’|E’ – W’|E), is the change in mean fitness owing to change in the environment. I now apply this partitioned Price Equation to the 2 models.

### The G × E Model

The definition of mean fitness for the G × E model is W = (1 + 2sf_1_p_A_). *After selection*, the mean fitness changes because the allele frequencies change, so that W’ now equals (1 + 2sf_1_pA’). (Note that, because f_1_ is the same before and after selection, the E’ = E, which makes the second term in Equation [7b] equal zero.) We can substitute (p_A_ + ∆p_A_) for p_A_’ and, using Equations [2a] and [5] above, we find that Equation [7a] becomes


$$\begin{array}{l} \Delta W_{GxE}=\;(W\text{'}|E\text{'})\;-\;\left(W|E\right) \end{array}$$ (8a)

$$\begin{array}{l} \Delta W_{GxE}=\;\{(1\;+\;2sf_{1}pA\text{`})\;-\;\left(1\;+\;2sf_{1}p_{A}\right)\}/W_{GxE} \end{array}$$ (8b)

$$\begin{array}{l} \Delta W_{GxE}=\;2sf_{1}\left(\Delta p_{A}\right) \end{array}$$ (8c)

$$\begin{array}{l} \Delta W_{GxE}=\;{\left(sf_{1}\right)}^{2}\left(2p_{A}q_{a}\right)/W_{GxE} \end{array}$$ (8d)

$$\begin{array}{l} \Delta W_{GxE}=\;V_{G}/W_{GxE}. \end{array}$$ (8e)

Fisher’s Fundamental Theorem is often interpreted as saying that “…*the rate of increase in mean fitness is equal to the genetic variance in fitness*” (Hartl and Clark 1989, p. 164), which is what we see in Equation [8e]. Walsh and Lynch (2018, chapter 14) say that “…*this interpretation of Fisher’s theorem holds exactly only under restricted conditions, but is often a good approximate descriptor*.” The Price Equation for G by E, where E is abiotic, illustrates that mean fitness changes over time but only by the first component, natural selection, and not by the second because there is no change in environment. Differently put, with G × E, the environment does *not* co-evolve with the gene frequency change. Lastly, I note in passing that the Opportunity for Selection (Wade 1979) equals (∆W_GxE_)/W_GxE_ which is V_G_/(W_GxE_)^2^, or the variance in *relative fitness*. This illustrates the direct connection between the Price Equation, Fisher’s Fundamental Theorem and the Opportunity for Selection.

### The G × G Model

The definition of mean fitness for the G × G model is W = (1 + 2sp_B_p_A_). *After selection*, the mean fitness changes because the allele frequencies at both loci change, so that W’ now equals (1 + 2p_B_’p_A_’).We can substitute (p_A_ + ∆p_A_) for p_A_’ and (p_B_ + ∆p_B_) for p_B_’, using Equations [4a] and [6] above, we find that Equation [7a] becomes


$$\begin{array}{l} \Delta W_{GxG}=\;(W\text{'}|E\text{'})\;-\;\left(W|E\right) \end{array}$$ (9a)

$$\begin{array}{l} \Delta W_{GxG}=\;\{(1\;+\;2sp_{B}\text{`}p_{A}\text{`})\;-\;\left(1\;+\;2sp_{B}p_{A}\right)\}/W_{GxG} \end{array}$$ (9b)

$$\begin{array}{l} \Delta W_{GxG}=\;2s\left(p_{B}\Delta p_{A}+\;p_{A}\Delta p_{B}+\;\Delta p_{A}\Delta p_{B}\right) \end{array}$$ (9c)

$$\begin{array}{l} \Delta W_{GxG}=\;V_{GA}/W_{GxG}+\;V_{GB}/2W_{GxG}+\;\left(V_{GxG}\right)/{\left(W_{GxG}\right)}^{2} \end{array}$$ (9d)

The change in W is different when the maternal genotypic environment co-evolves. The total change in mean fitness contains 3 terms: (1) V_GA_/W_GxG,_ the additive genetic variance; (2) V_GB_/2W_GxG_, the rate of change of the environment which equals the rate of change of the maternal B allele; and, (3) (V_GxG_)/(W_GxG_)^2^, the co-variance between offspring fitness and maternal environment.

(Note, that if there were no epistasis and only independent offspring and maternal effects, s and s_m_, the change in W would have contributions from the additive offspring variance owing to the A allele and to the additive maternal variance owing to the B allele; that is, the additive effects would contribute to the first term. This may be best seen in a single-gene model with *both* additive and maternal effects [e.g., Wade et al. 2009], where W = [1 + (s + [s_m_/2][2p_A_])] and V_G_ = (s + [s_m_/2])^2^ (2p_A_q_a_).)

Because the terms (2) and (3) are positive, evolution with this type of synergistic epistatic selection involving a genetic environment is *more rapid* than evolution with G × E where the environment is abiotic and does not evolve. Differently put, not only does the environment co-evolve, but the adapting offspring allele, A, becomes associated with the most adaptive maternal genotypic environment. Thus, the rate of evolution with G × G is very different than one might expect from an understanding of G × E.

A different interpretation of Equation [9d], based on Equations [6a] and [6b] is that the genic effects, (sp_B_) for the A locus and (sp_A_) for the B locus, tend to *increase* over time because each is a function of an increasing gene frequency (Figure 4). Moreover, epistatic selection creates a genetic covariance or LD between them.

## Discussion and Conclusion

Indirect genetic effects as heritable environments have important evolutionary consequences. Maternal genetic effects are likely *the* most common and important examples of indirect genetic effects. Previous models have studied 1-locus 2 allele models with indirect maternal effects and direct zygotic or offspring effects (e.g., Wade 1998; Wolf and Wade 2016; Fitzpatrick and Wade 2021, this volume) . These models have been extended to model evolution in variable environments (Dury and Wade 2020). Drown and Wade (2014) investigated 2-locus models with maternal-zygotic epistasis as well as other types of indirect-direct effect epistasis. This paper is an extension of Drown and Wade (2014) that uses the Price equation to better illustrate the contrast between adaptation to an abiotic environment and adaptation to a biotic environment. Many more cases with different types of indirect genetic effects, different types of fitness relationships among genotypes, and different mating systems remain to be explored. Here I have contrasted a model of maternal-zygotic epistasis, G × G _maternal_, and with a comparably parameterized model of G × E. Since a great deal more theoretical and empirical attention has been devoted to G × E, it provides a well-known basis for comparison. Moreover, I have modeled one of the simplest types of G × E, scale differences in genotypic fitness across environments. This type of G × E is often not a matter for practical concern because it can be eliminated, statistically, by changing the scale on which a phenotype was measured (James 2009; see also discussion of the Fisher-Hogben debate over G × E in Tabery 2015). As shown above, because the frequencies of environments in G × E models are often assumed to be constant, the second term in the Price equation remains 0.

The primary finding is that the rate of adaptive evolution with G × G_maternal_ can be much faster than the rate with G × E. The main reason for this difference is that the maternal environment is heritable and can co-evolve with an offspring gene adapting to it. In contrast, in G × E models, the underlying frequency of selective environments remains fixed throughout adaptive evolution. There is a positively synergistic interaction between maternal and zygotic genes that causes the offspring genic effect on fitness, sp_B_, to change at each generation as the B allele in the maternal background increases in frequency. Differently put, whatever the statistical merit of transforming away scale type G × E when the environment is fixed, it is much more difficult to ignore or minimize the importance of such interactions when the scale effect itself is evolving and changing at every generation.

There are many other types of fitness relationships between maternal and direct genetic effects that could be modeled. Often, in natural systems, a negative genetic correlation is observed between maternal and direct effects, possibly owing to natural selection favoring an intermediate phenotype although there are other causes of negative correlations (cf. review by Lee 2002). These cases may not behave in the same way as as the case examined here. Similarly, epistasis is itself a controversial subject in evolutionary biology: “*Contrasting views of the genetic architecture underlying fitness-related traits have polarized evolutionists since Darwin’s time”* (Fenster et al. 1997; see also Crow 2010 and Hill et al. 2008). However, a recent investigation of long-term protein evolution for proteins involved in mitochondrial-nuclear interactions concluded that (Breen et al. 2012) “…*about 90 per cent of all amino-acid substitutions have a neutral or beneficial impact only in the genetic backgrounds in which they occur, and must therefore be deleterious in a different background of other species*.” It is one of the interesting features of additive × additive epistasis for fitness that the sign of an allele’s effect on fitness changes as the genetic background at another locus changes (see Wade 2002 for an analysis of all 4 types of 2-gene epistasis). Maternal-zygotic interactions have not yet been subject to such gene-level empirical studies, although the genomic study of indirect genetic effects has begun (e.g., Brinker et al. 2018). By partitioning the variance in fitness into its components, I have illustrated the quantitative parallels between the 2 models (compare Equations [2] and [4]). Despite the parallel structure, V_G_ component of fitness variation behaves very differently in the G × G_maternal_ model than it does in the G × E model. Using the Price Equation, I showed how, in both models, the genetic variance affects the rate of increase in W, mean fitness, as expected from Fisher’s Fundamental Theorem. However, there is a second component to the change in W that appears in the G × G model that is absent in the G × E model. Here, it proves to be as important as the familiar first component, the additive genetic variance in fitness, and, in other cases, it may be more or less important, depending upon the nature of the fitness inter.
